# Editorial: Supramolecular Metal-Based Entities for Biomedical and Biological Applications

**DOI:** 10.3389/fchem.2019.00293

**Published:** 2019-04-26

**Authors:** Angela Casini, James D. Crowley

**Affiliations:** ^1^School of Chemistry, Cardiff University, Cardiff, United Kingdom; ^2^Department of Chemistry, University of Otago, Dunedin, New Zealand

**Keywords:** supramolecular complex, MOF (Metal-Organic-Framework), metallacages, metallacycle complexes, therapy, drug delivery systems, cancer, self-assembly

The biomedical and biological application of supramolecular metal-based structures—including the discrete supramolecular coordination complexes (SCCs) and polymeric metal organic frameworks (MOFs)—is an emergent research area. In fact, in the last decade, a number of studies demonstrated the potential of these supramolecular compounds as novel anticancer drugs, drug delivery systems, and imaging agents, as well as for molecular recognition of relevant biomolecules.

The present article collection includes an overview of the examples of discrete 2D- and 3D-supramolecular metal-based systems, with focus on cancer therapy, in the review of Ahmedova. The review covers the specific case of 2D metalacycles, as well as 3D metallacages, helicates, capsules and barrels, and marks out some key tendencies in relation to their biological activity and potential areas of biomedical application.

Of note, the point of view of one of the pioneers in this research area, Therrien, is provided in a review focused on the role of the second coordination sphere, including H-bonding, hydrophobic, electrostatic, and van der Waals interactions, in the biological activity of arene ruthenium metalla-assemblies. These types of interactions are crucial in the design of supramolecular entities implemented for molecular recognition.

Thus, a number of original papers in this issue report on the use of selected metallacages as cytotoxic agents against human cancer cells. For example, Vasdev et al. reported on [Pd_2_L_4_]^4+^ metallacages featuring *bis*-quinoline and bis-isoquinoline-based ligands. The *bis*-quinolone-based cage was found to be helical while the *bis*-isoquinoline-based system was lantern shaped. The binding of the anticancer drug cisplatin was also examined. It was shown that only the *bis*-isoquinoline cage interacted with the drug, suggesting that the helical twist interferes with the host-guest interaction. Both new cages were shown to be more cytotoxic against cancer cell lines than related pyridyl based systems.

Interestingly, Wang et al. provided the proof-of-concept of using nanoprecipitation to formulate hydrophobic Pt(II)-organic cages.

Along the same line, the antiproliferative effects of a self-assembled hexagonal Pd(II) macrocycle against a small panel of cancer cell lines were found comparable to cisplatin in the paper by Singh et al.

Furthermore, Sun et al. explored the potential of encapsulating Ru(II) polypyridyl complexes in macrocyclic host compounds, namely cucurbit[10]urils (Q[10]), to reduce the toxicity of Ru(II) complexes. The Q[10] encapsulation enhanced the pharmacokinetics of one of the ruthenium complexes which could potentially enable the complex to be used as a new type of antimicrobial agent. The host-guest complex between twisted cucurbit[14]uril and thioflavin T was also explored for sensing of non-fluorescent triazole fungicides by Fan et al.

Peptides are highly versatile building blocks, encoding precise structural and functional information within their amino acid sequence, which can be used to design and synthesize novel supramolecular metal-based helicates structures endowed with molecular recognition properties. In the work by Gómez-González et al. the modification of the T4 Fibritin foldon with metal-chelating bipyridines allowed the obtainment of Fe helicates which display good *in vitro* DNA binding and selectivity toward three-way DNA junctions.

Among the features of supramolecular-based scaffolds, their host-guest chemistry is certainly interesting to exploit them as drug delivery systems or as biosensors. Thus, Woods et al. reported on a series of *exo*-functionalized [Pd_2_L_4_]^4+^ metallacages featuring 2,6-bis(pyridin-3-ylethynyl)pyridine) ligands. The encapsulation of the anticancer drug cisplatin in selected cages has been studied using a combination of NMR spectroscopy methods, and the obtained results show that if the solvent is of sufficient polarity, cisplatin encapsulation can easily occur in the hydrophobic cavity of the cage despite the absence of hydrogen-bond accepting cavity-facing residues.

A novel aspect of this research field concerns the use of supramolecular organometallic complexes, whereby the (organic) linker is forming the organometallic bond to the metal node, for therapy. Thus, the antimicrobial and anticancer properties of organometallic Ag(I) and Au(I) pillarplexes were explored in different bacterial and human cancer cells, respectively (Pöthig et al.). The obtained results showed more relevant bioactivity in the case of the silver compounds.

Within this framework, Barnard et al. developed the synthesis of Au(I) and Pd(II) N-heterocyclic carbene (NHC) complexes derived from tetra-imidazolium linked macrocycles for anion sensing. Interestingly, some of the Ag(I) and the analogous Au(I) complexes adopted intriguing hexanuclear structures with the general formula [M_6_L_3_]^6+^. It is envisaged that these novel scaffolds could allow the slow release of metal ions for the treatment of bacterial infections.

Overall, as highlighted in the contributions to this special issue, the myriad of possible metal-based supramolecular structures and their almost limitless modularity and tunability suggests that the biomedical applications of these complex chemical entities will continue to emerge.

## Author Contributions

All authors listed have made a substantial, direct and intellectual contribution to the work, and approved it for publication.

### Conflict of Interest Statement

The authors declare that the research was conducted in the absence of any commercial or financial relationships that could be construed as a potential conflict of interest.

